# Patent Foramen Ovale—A Not So Innocuous Septal Atrial Defect in Adults

**DOI:** 10.3390/jcdd8060060

**Published:** 2021-05-25

**Authors:** Veronica Romano, Carlo Maria Gallinoro, Rosita Mottola, Alessandro Serio, Franca Di Meglio, Clotilde Castaldo, Felice Sirico, Daria Nurzynska

**Affiliations:** 1 Department of Public Health, University of Naples “Federico II”, 80131 Naples, Italy; veronica.romano@unina.it (V.R.); carlomaria.gallinoro@unina.it (C.M.G.); rosi.mottola@studenti.unina.it (R.M.); alessandro.serio@unina.it (A.S.); franca.dimeglio@unina.it (F.D.M.); clotilde.castaldo@unina.it (C.C.); 2 Department of Medicine, Surgery and Dentistry “ScuolaMedicaSalernitana”, University of Salerno, 84081 Baronissi, Italy

**Keywords:** foramen ovale, patent foramen ovale, atrial septal defect, diving, decompression illness

## Abstract

Patent foramen ovale (PFO) is a common congenital atrial septal defect with an incidence of 15–35% in the adult population. The development of the interatrial septum is a process that begins in the fourth gestational week and is completed only after birth. During intrauterine life, the foramen ovale allows the passage of highly oxygenated blood from the right to the left atrium and into the systemic arteries, thus bypassing the pulmonary circulation. In 75% of the general population, the foramen ovale closes after birth, and only an oval depression, called fossa ovalis, remains on the right side of the interatrial septum. Patent foramen ovale can be associated with various clinically important conditions, including migraine and stroke, or decompression illness in divers. The aim of this review is to summarize the PFO developmental and anatomical features and to discuss the clinical risks associated with this atrial septal defect in adults.

## 1. Introduction

Patent foramen ovale (PFO) is a common defect of the interatrial septum, with an incidence of 15–35% in the adult population [1]. During intrauterine life, the existence of the foramen ovale allows the placental oxygen-rich blood entering the right atrium to be shunted into the fetal arterial systemic circulation. During fetal life, the lungs are collapsed and filled with amniotic fluid, the pressure in the pulmonary circulation is low, and the amount of blood required is low [2]. The placental oxygenated blood reaches the right atrium through the inferior vena cava, where it mixes with a small amount of poorly oxygenated blood coming from the venae cavae, and is introduced through the foramen ovale into the left atrium and then ventricle, thus bypassing the pulmonary circulation [3]. Once the oxygenated blood has reached the left ventricle, it will preferentially be directed into the coronary arteries and the carotids, while only a small amount will be directed into the descending aorta, thus favoring a greater development of the heart, brain, and head than the rest of the body.

When pressures in the systemic and pulmonary circulation increase after birth, the greater pressure in the left atrium allows the closure of the foramen ovale by coalescence of the septum primum and septum secundum, producing a depression called fossa ovalis on the right atrial surface [1,3]. In 15–35% of the population, it does not occur and the foramen ovale persists. In most cases, the PFO is completely asymptomatic. In some subjects, however, it can be associated with stroke due to paradoxical embolization [1,4,5,6,7,8,9], migraine with aura [10], decompression sickness [11,12], or hypoxemic medical conditions, such as, sleep apnea, chronic obstructive pulmonary disease, and pulmonary hypertension [13,14].

## 2. Development of Interatrial Septum and Foramen Ovale

The heart begins to develop from the splanchnic mesoderm of the first heart field, which gives origin to two heart tubes that merge to form a single primitive heart tube. It is composed of a layer of myocardium and a layer of acellular matrix, called cardiac jelly, lined by endocardium. In the early stages of development, the primitive heart tube is attached to the pharyngeal mesoderm by a dorsal mesocardium. The pharyngeal mesoderm, indeed, will give origin to the second wave of cardiac progenitor cells (second heart field), which contribute to the elongation of the heart tube at its poles and the formation of the right ventricle wall [15]. During the fourth gestational week, as the heart tube begins to loop, most of the dorsal mesocardium will disappear, leaving a dorsal mesenchymal protrusion at the cardiac venous pole [16]. With the onset of ballooning, leading to the partitioning of the atrioventricular canal, the cardiac jelly protrudes at the ventricular outflow tract and at the atrioventricular junction. The atrioventricularendocardial cushions, formed at the latter location as a result of epithelial–mesenchymal transition, contribute to the formation of the valves and membranous parts of the cardiac septa [17].

**Figure 1:**
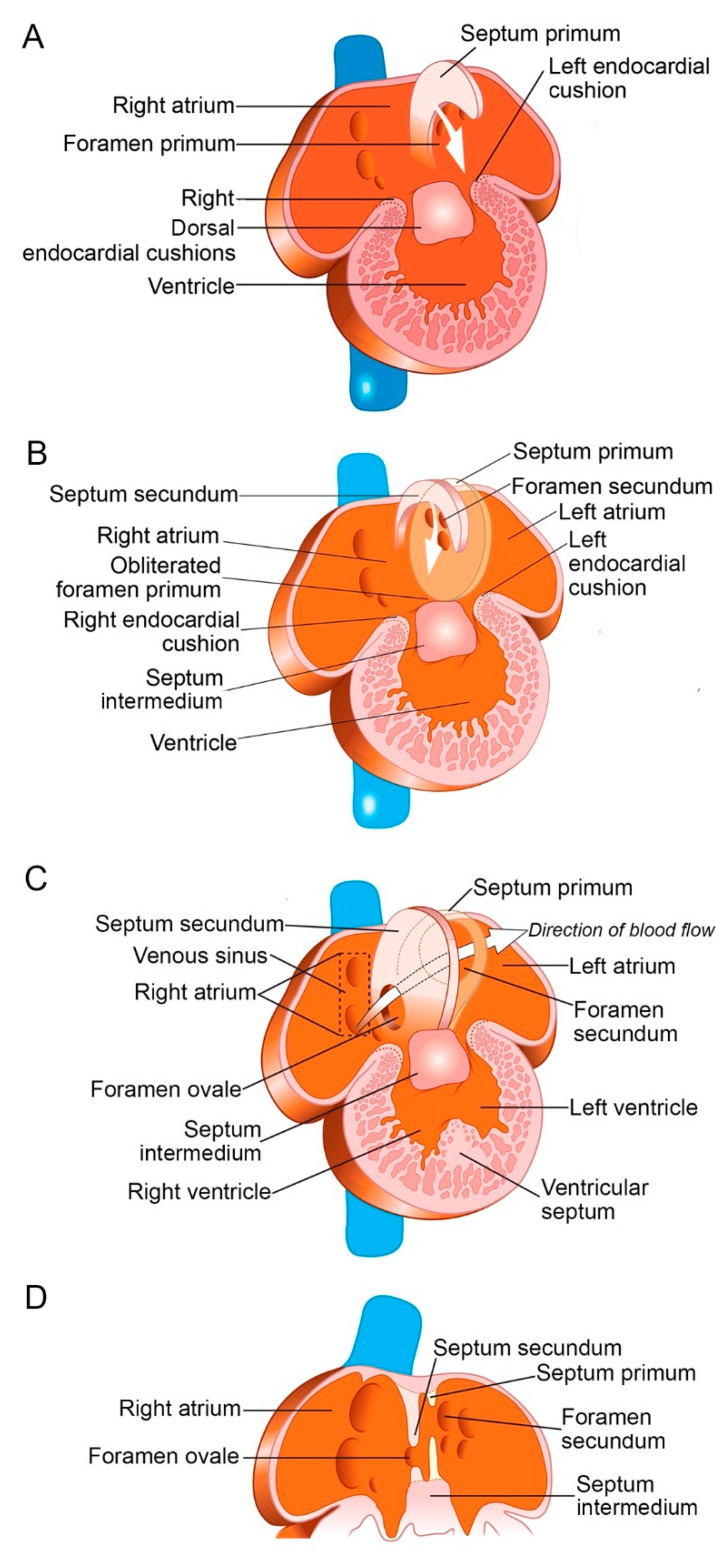
Development of the interatrial septum and foramen ovale at the end of the fourth week (**A**), during the sixth week (**B**), and at the beginning of the seventh gestational week (**C,D**). Modified with permission [22].

Directly prior to the partitioning of the primordial atrium, the heart detaches from the dorsal mesocardium, and the cardiac jelly of the atria begins to retract so that the endocardium adheres to the myocardium, except at a small ridge of mesenchyme that crosses the atrial roof from the ventral part of the atrioventricular cushions to the dorsal mesenchymal protrusion [18]. This mesenchymal bridge, with a cap expressing the transcription factor Tbx3 at the leading edge, grows caudally towards the atrioventricular cushions to form the primary septum [19]. The gap between the atrioventricular cushions and the leading edge of the septum primum is the primary foramen (or foramen primum), which shrinks as the septum grows (Figure 1A).

Around the fifth gestational week, the mesenchymal cap arrives in contact with the atrioventricular cushions, and the growth of the septum primum terminates. At the same time, however, the cranial part of the septum primum develops one or several fenestrations, which coalesce to form the secondary foramen (or foramen secundum, Figure 1B). This ensures right-to-left shunting of oxygenated blood that would have been otherwise impossible with the closure of the septum primum. During fetal life, this shunt to the left atrium is responsible for about 20–30% of the total cardiac output.

During the sixth week, a folding of the right atrial roof begins between the primary septum and the ostium of the superior vena cava, which represents the first step of the development of the dorsocranial margin of the septum secundum, gradually overlapping the foramen secundum in the septum primum (Figure 1B). During the last 20 weeks of gestation, fetal growth occurs very quickly; nonetheless, the foramen ovale, which now includes a gap between the primary septum and the secondary septum, remains proportional to the size of the right atrium (Figure 1C) [20]. The upper part of the septum primum degenerates, while its lower part acts as a one-way flap, called valve of the foramen ovale (Figure 1D). In fact, as can be seen by echocardiography, this flap closes the foramen ovale for about 20% of the cardiac cycle duration [21].

Occasionally, the foramen ovale closes during intrauterine life; this results in significant hypertrophy of the right atrium and ventricle, which can lead to arrhythmias, pleural effusion, right heart failure, ascites, and hydrops. Death usually occurs shortly after birth [20].

## 3. Foramen Ovale after Birth

After birth, the pressure of the pulmonary circulation drops, the pressure of the systemic circulation increases, and this pressure gradient causes the adhesion of the valve of the foramen ovale to the septum secundum [17]. In 75% of the general population, the foramen ovale closes within a few months, and only an oval depression, called fossa ovalis, remains on the right side of the interatrial septum, slightly above and to the left of the orifice of the inferior vena cava [1]. It is surrounded by a raised muscular rim, called limbus fossae ovalis, and its floor is formed by the septum primum. The raised fossa flap is a fold in the walls of the atrial chambers that incorporates fatty tissue. The interatrial septum seen from the left atrium is devoid of particular features [21].

**Figure 2:**
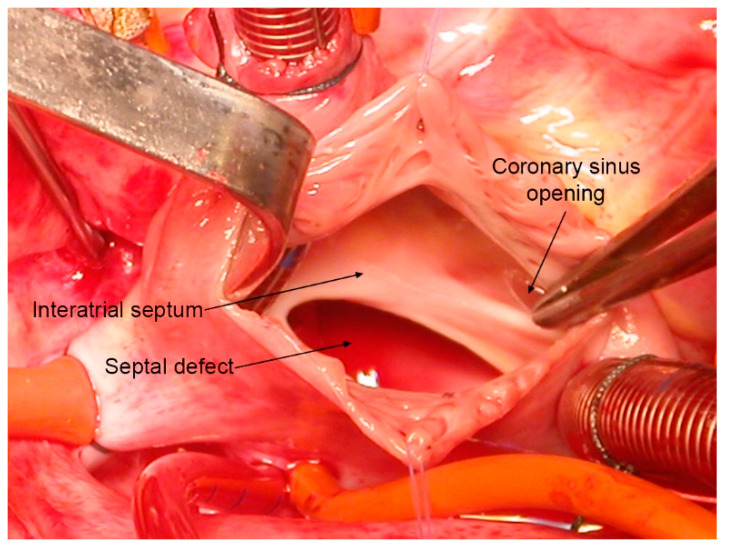
Intraoperative view of an atrial septal defect, ostium secundum type. This defect can result from excessive resorption of the proximal part of the septum primum or the inadequate development of the septum secundum [1].

In 25% of the population, however, foramen ovale remains open and allows a small shunt of poorly oxygenated blood from the right to the left circulation [23] (Figure 2). The prevalence of the PFO is the same in males and females. Some studies suggest a race–ethnic difference in the prevalence and size of the PFO [24], but this observation still needs to be confirmed by systematic analysis. In most people with PFO, the amount of venous blood shunted to the left atrium has no clinical significance [20]. As will be discussed later, in certain concomitant conditions or during particular activities, the presence of the foramen ovale can have fatal consequences.

## 4. Anatomy of PFO

The PFO is a small defect at the upper edge of the fossa ovalis that continues upwards, underneath the fossa flap, and communicates with the left atrium. In autopsy studies, the size of PFO in adult hearts varies from 1 to 19 mm. Importantly, it has been observed that the mean size increases with each decade of life [23]. 

Two PFO variants have been described. The first, valve competent, has a perfect seal, created by the septum primum that perfectly overlaps the PFO muscular margin and acts as a valve of the foramen ovale. In physiological conditions, this valve prevents any shunting of blood between the atria. In some cases, there is an aneurysmal valve that can bend towards one of the atria during breathing movements. The second variant is valve incompetent, as there is no adequate overlap between the valve of the foramen ovale and the PFO margin. This variant may be secondary to a retraction of the aneurysmal valve, an increase in the size of the atrium, a defect in the muscular rim, or a primary defect due to excessive resorption of the septum primum or inadequate development of the septum secundum [20].

## 5. Etiology of PFO

Approximately 25% of congenital heart defects occur as a component of a syndrome, with malformation of other organs. In the remaining 75% of cases, cardiac defects are isolated; most of these cases are sporadic, but some are familial and can follow a Mendelian inheritance pattern [25].

Both environmental and genetic factors have been associated with congenital heart defects, such as PFO. Known risk factors include maternal exposure to polychlorinated biphenyls and pesticides [26], alcohol, cocaine and rubella [27], retinoic acid during the first trimester, antiretroviral medication, and maternal diabetes [28]. As regards genetics, the well-known association of congenital heart defects with chromosomal aneuploidy (Down syndrome) or intragenic mutation (Noonan syndrome) is discussed elsewhere [25]. Moreover, PFO was found to be associated with mutations in genes coding for structural proteins, such as cardiac myosin heavy chain (MYH6) [29], and for transcription factors involved in cardiac development: Nkx2.5 (involved in tetralogy of Fallot and electrical conduction disorders) [30], TBX5 (causing heart–hand syndrome with septal defects, dysrhythmias, and upper limb malformations) [31], TBX20, and GATA4 [32]. These genes, however, have been implicated only in a small proportion of nonsyndromic asymptomatic PFO in adults.

## 6. Diagnosis of PFO

**Figure 3:**
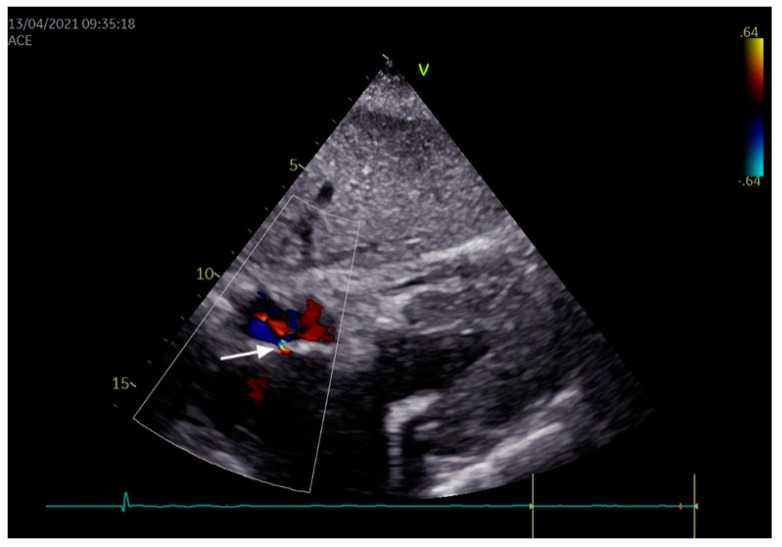
An example of patent foramen ovale, as observed in the subcostal view using color Doppler. The arrow indicates a small left-to-right atrial shunt.

With the advent of new diagnostic imaging techniques, such as transthoracic echocardiography with bubble test, transesophageal echocardiography, and transcranial Doppler ultrasound, the confirmation of the PFO has become clinically attainable (Figure 3). The transthoracic echocardiography with bubble test is a noninvasive exam that detects PFO with 99% specificity and 46% sensitivity. Unlike traditional echocardiography, this test is performed after injecting an agitated saline contrast (i.e., a solution capable of forming microbubbles) into the patient’s peripheral vein. The presence of PFO is suggested by the observation of saline microbubbles shunting to the left atrium in at least three cardiac cycles, especially after Valsalva maneuver [33]. The main limitations of such test are its low sensitivity and lack of detailed information on interatrial septal defect morphology [34].

Transesophageal echocardiography is a semi-invasive exam with 92% specificity and 89% sensitivity; however, it requires patient sedations that preclude the use of provocative maneuvers. Hence, it is used after a negative agitated saline echo testing when the high suspicion for PFO remains or when a better anatomic definition of atrial septum is needed. Transesophageal echocardiogram can show right-to-left shunt both with echo-color Doppler and after administration of an intravenous contrast agent [11]. 

**Figure 4:**
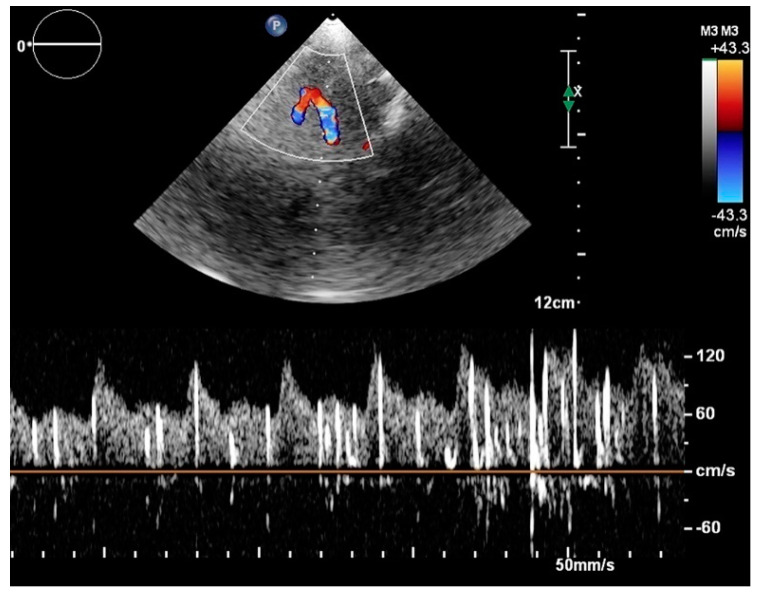
An example of transcranial Doppler ultrasound with the agitated saline contrast, in which the microbubbles produce embolic signal within the middle cerebral artery.

Transcranial Doppler ultrasound (Figure 4) is just as specific as the previously described techniques and even more sensitive (96%) than transesophageal echocardiography [33]. It examines the blood circulation within the brain by monitoring middle cerebral arteries through the temporal window using 2-MHz probes after the injection of the agitated saline contrast agent [35]. In the presence of the atrial septal defect and right-to-left atrial shunt, the contrast solution bypasses the pulmonary circulation and causes microembolic signals in the cerebral arteries, which are detected by transcranial Doppler ultrasound [36]. This approach offers little information on the anatomy of the atrial septum defect; nonetheless, it has an excellent diagnostic accuracy and is recommended as a first-choice screening tool for PFO in patients with cryptogenic stroke [37].

## 7. Clinical Features of PFO

Most people with PFO remain asymptomatic throughout life, but life-threatening events can develop in association with certain pathologies or activities, including cryptogenic stroke, migraine with aura, or decompression sickness in divers.

Cryptogenic stroke can be defined as ischemic stroke of undetermined origin [38]. People with PFO have only 0.1% risk of a first stroke without other concomitant cause, but several studies have shown that PFO is highly prevalent (up to 46%) in cryptogenic stroke in young adults [9]. A cerebrovascular accident, in such cases, can be caused by paradoxical embolism (i.e., an embolus that has formed in a peripheral vein or in the right atrium and has reached the cerebral circulation through the PFO) [39]. 

The prevalence of PFO in people with migraine reaches 50%, and migraine with aura occurs more frequently in patients with PFO than in the general population [40]. The correlation between PFO and migraine is suggested by the theory that migraine can be triggered by the passage of serotonin or other factors from the right to the left atrium, hence avoiding the metabolic transformation in the lungs and gaining entry to the systemic circulation at a higher concentration, so that on reaching the brain, they can stimulate receptors in susceptible individuals. Another theory suggests that microemboli from the venous circulation can pass through the PFO, causing ischemia, cortical irritability, and subsequently depression and/or migraine [41,42].

According to the recently published guidelines on the management of patients with PFO [6], the decision to treat and the choice of treatment require collaboration between an interventional cardiologist and a neurologist or other relevant specialists. The risk of recurrence in PFO-associated stroke is quite low, and there are yet no clear recommendations regarding the choice of secondary prevention (antiplatelet therapy, oral anticoagulation, or percutaneous closure). 

## 8. Diving with PFO

Over the last 40 years, interest in the close link between PFO and diving has grown rapidly, as it has been observed that the PFO can cause paradoxical embolization of nitrogen bubbles that form during diver ascent [43]. Nowadays, this cardiac malformation is considered a risk factor for the onset of decompression sickness, one of the most feared complications of diving. 

During diving, the pressure on the diver’s body increases with depth. According to Henry’s law, as pressure grows, the solubility of gases in a liquid increases. Thus, during underwater descent, more gas dissolves in the diver’s blood and tissue interstitial liquid. The gas mixtures breathed in by professional divers vary depending on the program, but most of them contain a large percentage of nitrogen. The oxygen contained in the mixture is consumed by cellular metabolism, while nitrogen accumulates in the blood and tissues. During return to the water surface, a decrease in pressure acting on the body determines a reduction in the solubility of nitrogen with a formation of microbubbles, which then reach the lungs and can be eliminated by breathing out. In the case of a too rapid ascent, if the diver violates the decompression protocol, or at times even if all the procedures are carried out correctly, it may happen that the decompression occurs too quickly, causing the excessive formation of bubbles that can bring about tissue damage or vascular embolism. This clinical phenomenon is called decompression illness (DCI) [12]. 

The DCI includes two pathological entities: arterial gas embolism (AGE) and decompression sickness (DCS, types I and II). AGE occurs immediately after ascent and is often caused by errors in the return to the surface and the presence of pre-existing lung pathologies associated with bullae. These factors determine the stretching and subsequent laceration of the alveoli (barotrauma) with consequent introduction of air bubbles first into the pulmonary veins and then into the arterial circulation.

DCS occurs about 20 min after rising to the surface and is caused by nitrogen bubbles formed during ascent and reaching a concentration that exceeds the capacity of the tissues and the venous circulation to eliminate their excess. So-called minor DCS (i.e., DCS type I) is caused by the local formation of bubbles and presents with skin and/or musculoskeletal symptoms, such as itching, skin rash, and joint pain. Major DCS, or type II, is due to the formation of venous bubbles that induce neurological, vestibular (dizziness/vertigo), or pulmonary symptoms in the presence of a right-to-left shunt caused by PFO [44]. 

Epidemiologically, about 10% of divers experience symptoms of DCS related to their diving activity [45]. Divers with PFO present greater risk of DCS, as the presence of a right-to-left shunt allows for the paradoxical embolization of gas bubbles formed during ascent to the surface, resulting more often in DCS type 2. Several studies suggest that in divers with PFO, the risk of DCS increases up to six times (in relation to the size of the septum defect), and the risk of neurological DCS is four times higher than normal [45,46,47]. Several factors are known to play a role in increasing the risk of DCS in these divers, apart from errors in applying the decompression protocol [12,48]. First, the Valsalva maneuver, used to compensate differences between ambient pressure and middle ear pressure, increases the pressure in the right part of the heart, thus increasing the right-to-left shunt [48,49]. Second, during ascent to the surface, the blood from the lower limbs is drawn into the chest, contributing to the increase of pressure in the right heart [49].

According to recent recommendations, there is no need for routine screening for PFO in divers [50]. An evaluation should be performed only in case of DCS type 2 in the past, current, or past history of migraine with aura or history of cryptogenic stroke. If PFO is diagnosed, the diver should consider three possible strategies: stop diving, practice conservative diving, or perform PFO closure. Several studies have shown that conservative diving reduces the risk of DCS through the adoption of specific measures, including reduction the depth of the dive, reduction of the number of descents to only one dive per day, and increase of the duration of ascent steps implemented in the decompression protocols [45,51,52]. The percutaneous closure of PFO, however, remains the most effective strategy in patients who have already experienced DCS and want to fully continue the immersive activity [12,45,49,53]. After PFO closure, the risk of DCS is comparable to that of the normal population, while the risk of AGE is unaffected [54]. The choice of PFO percutaneous closure must take into account the risk–benefit ratio of the procedure, with complications such as cardiac tamponade (0.4–0.7%), device embolization (0.7%), and bleeding (0.4%). Henzel et al. [55] estimated that the risk of DCS and PFO closure intervention is equalized after 100–200 dives, and this procedure would therefore be recommended in regular divers. Only divers in whom complete closure has been confirmed should be allowed to return to “unrestricted” diving [50].

## 9. Take-Home Message

PFO is present in up to 35% of adults. Often clinically silent and of little hemodynamic significance, it still needs to be considered in patients with migraine or stroke or individuals involved in certain hemodynamically challenging activities, such as diving.
